# Clinicopathologic implication of meticulous pathologic examination of regional lymph nodes in gastric cancer patients

**DOI:** 10.1371/journal.pone.0174814

**Published:** 2017-03-31

**Authors:** Jiwon Koh, Hee Eun Lee, Woo Ho Kim, Hye Seung Lee

**Affiliations:** 1 Department of Pathology, Seoul National University College of Medicine, Seoul, Republic of Korea; 2 Department of Laboratory Medicine and Pathology, Mayo Clinic, Rochester, MN, United States of America; 3 Department of Pathology, Seoul National University Bundang Hospital, Seongnam, Republic of Korea; University of Texas MD Anderson Cancer Center, UNITED STATES

## Abstract

**Background:**

We aimed to investigate effect of increased number of examined lymph nodes (LNs) to pN category, and compare various N categories in gastric cancer: American Joint Committee on Cancer (AJCC) 7^th^ edition, metastatic LN ratio (MLR), and log odds of positive LNs (LODDS).

**Methods:**

Four cohorts with a total of 2,309 gastric cancer patients were enrolled. For cohort 1 and 2, prognostic significance of each method by disease-specific survival was analyzed using Akaike and Bayesian information criterion (AIC and BIC).

**Results:**

The total LNs in four cohorts significantly differed [median (range), 28 (6–97) in cohort 1, 37 (8–120) in cohort 2, 48 (7–122) in cohort 3, and 54 (4–221) in cohort 4; p<0.001]. The numbers of negative LNs increased with increase of total LN (p<0.001), but the numbers of metastatic LNs did not increase from cohort 1 to 4. MLR and LODDS in four cohorts had decreasing tendency with increase of total LNs in each pT3 and pT4 category (p<0.001), while the numbers of metastatic LNs did not differ significantly in any pT category (p>0.05). The AIC and BIC varied according to different cut-off values for MLR; model by cut-offs of 0.2 and 0.5 being better for cohort 1, while cut-offs 0.1 and 0.25 was better for cohort 2.

**Conclusion:**

Our study showed that the number of metastatic LNs did not increase with maximal pathologic examination of regional LNs. AJCC 7^th^ system is suggested as the simplest method with single cut-off value, but prognostic significance of MLR may be influenced by various cut-offs.

## Introduction

Gastric cancer (GC) is one of the most common types of cancer and the leading causes of death, accounting for 10% of total cancer-associated deaths worldwide [1]. In South Korea, about 35,000 people are newly diagnosed with GC annually, and it is the third most common cause of cancer mortality [2]. Lymph node (LN) involvement has long been considered to be the most important prognostic factor in GC [3]. The AJCC 7^th^ edition staging system uses the absolute number of positive LNs to assess the N status, which has long been accepted as the routine way of evaluating regional LN status [4,5].

There has been criticism against the requirement of the AJCC 7^th^ edition N-category system–that optimal specimens should contain at least 16 regional LNs [6]. While Asian countries including Korea and Japan routinely practice D2 dissection of LNs, Western countries take more conservative stance on LN dissection, D1 dissection being the most popular [7]. Therefore, Western surgical oncologists and pathologists have difficulty in harvesting more than 15 LNs. For instance, a study performed in 2006 using the Surveillance Epidemiology and End Results (SEER) database revealed that among the 10,807 surgically resected GC cases, only 29% met the minimal requirements [8]. Recent studies have demonstrated that the number of total or negative LNs could predict patients’ prognosis [9] and that insufficient number of examined LNs could be a cause of inaccurate prediction of patients’ outcome [10]. Therefore, surgeons usually demand maximal retrieval of LNs on pathologists, but the clinical significance of meticulous pathologic LN retrieval after standardized surgical procedure has not been clear. There has been another major criticism against the number-based AJCC N- category system: stage migration. The term stage migration refers to the phenomenon where a lower number of examined LNs results in understaging of N-status [11], while a higher number of nodes causes unnecessary overstaging [12,13].

The alternative N- category methods which can be utilised regardless of the total number of retrieved LNs were suggested, and most well-known methods include metastatic LN ratio (MLR) [14] and log odds of positive LNs (LODDS) [15]. The MLR is defined as the ratio of the number of metastatic LNs to the total number of examined LNs. It is known for its flexibility in various clinical situation: D1 or D2 dissection, and LNs less than 15 or more than 16 [16]. Although there are still ongoing debates regarding the cut-off values of MLRs, recent studies state that MLR has less influence on stage migration [14] and is more accurate in the prediction of prognosis [17].

Some researchers raised concerns for the pN0 categories of both the AJCC 7^th^ edition and MLR systems; they suggest that the pN0 category, of which the proportion is around 40%, may not be a homogeneous category [18]. Both systems are not able to discriminate the prognoses of patients within N0 category [19]. Therefore, to prevent all node-negative patients from being categorized into a single N0 category, the LODDS method was proposed as $\log\;\frac{\left(\text{positive node}+0.5\right)}{\left(total\;node-positive\;node+0.5\right)}$ [20], and the generated values were stratified by certain cut-off values into LODDS. The supporters of the LODDS system assert that this method provides a better prediction of prognosis, however, the complexity of the formula has consistently been at the center of criticism.

Accurate analysis of LN status in GC is of crucial importance for predicting the prognosis. Therefore, we aimed to investigate clinical implication of maximal retrieval of LNs in pathologic laboratories and to compare the three aforementioned types of LN assessment methods to determine the most useful model.

## Materials and methods

A total of 2,309 patients who had been diagnosed with GC and consecutively undergone surgical resection at two individual institutions were included in this study. 664 patients were treated at Seoul National University Hospital (Seoul, Republic of Korea) in 2004 (cohort 1). The remaining 1,645 patients were treated at Seoul National University Bundang Hospital (Seongnam, Republic of Korea): 579 patients between January 2003 and December 2005 (cohort 2) and 587 and 479 patients in the years 2011 (cohort 3) and 2013 (cohort 4), respectively. The cases consisted of primary and sporadic GCs; recurred, metastatic, or hereditary cancers were excluded. None had received preoperative chemotherapy or radiotherapy. The patients with stage II to IV disease received adjuvant chemotherapy using fluoropyrimidine (5-fluorouracil, capecitabine, or S-1) alone or fluoropyrimidine plus mitomycin C, cisplatin, or oxaliplatin, if clinically indicated.

Clinicopathologic data were collected retrospectively from medical records and pathologic reports. Clinical outcomes were followed from the date of surgery in cohort 1 and 2, and sufficient follow-up time (1–109 months (median, 53 months)) was provided. Cases lost to follow-up and deaths by causes other than GC were censored. Disease specific survival was defined as the time between the date of surgery and the date of death of gastric cancer-related cause or last follow up date. When the dates and causes of patients’ death were checked by the legitimate database from the Ministry of Public Administrations and Security in Korea; if the relevant data were not available from the governmental database, we reviewed the medical records for additional information. The pN stage by AJCC 7^th^ edition was categorised, and for MLRs, since there was no consensus on cut-off value, we adapted the three sets of cut-off values that have been used most frequently in previous studies; 0.3 and 0.6 [20], 0.2 and 0.5 [14], and 0.1 and 0.25 [21]. LODDS was divided into four stages according to the most commonly used cut-off values: pLODDS1 (LODDS ≤ −0.5), pLODDS2 (-0.5 < LODDS ≤ 0), pLODDS3 (0 < LODDS ≤ 0.5), pLODDS4 (0.5 < LODDS) [20].

This study was approved by the institutional review board of Seoul National University Hospital and Seoul National University Bundang Hospital (IRB number: B-1507/306-115). All medical and pathologic records were anonymized before use in this study. The participants did not provide written informed consent, but the institutional review board waived the need for written informed consent under the condition of anonymization and no additional intervention to the participants.

The chi-square test or Fisher’s exact test was performed to analyse categorical variables. The total number of retrieved LNs, number of negative LNs, number of metastatic LNs, MLR, and LODDS were non-parametric variables by tests of normality (p < 0.001 by Kolmogorov-Smirnov or Shapiro-Wilk tests), and these were compared using the Kruskal-Wallis method among four cohorts and the Mann Whitney U method between the two cohorts. The *p*-values < 0.5 were considered statistically significant. For the 1^st^ and 2^nd^ cohorts, the disease-specific survival (DSS) and Akaike information criterion (AIC) and Bayesian information criterion (BIC) indices were obtained to compare each N- category model [22], AIC model was based on Cox proportional hazard model. All statistical analyses were performed with the SPSS Statistics 21.0 software package (SPSS Inc., Chicago, IL, USA) except for AIC and BIC calculation which were performed using the R statistical package 3.1.1 (http://www.r-project.org).

## Results

### Clinical features and LN status in four cohorts

**Fig 1:**
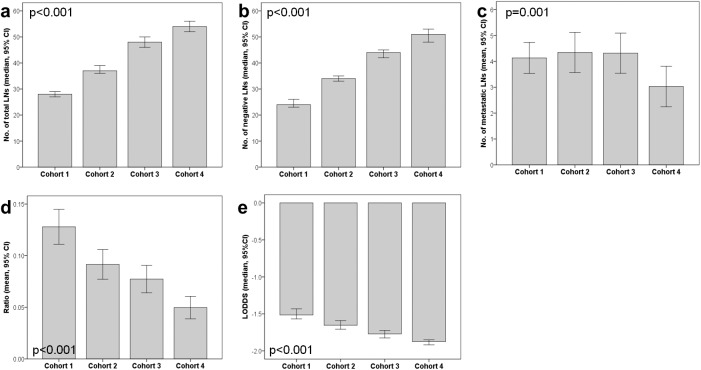
Numbers of total LNs (a), numbers of negative LNs (b), numbers of metastatic LNs (c), metastatic lymph node ratio (MLR) (d), and log odds of positive lymph nodes (LODDS) (e) among four cohorts were plotted by bar charts and analyzed by Kruskal-Wallis H tests

The clinicopathologic characteristics of the four cohorts are summarized in Table 1. The median age (range) was 61 years (23–89), and cohort 1 was younger than other cohorts (p < 0.001). There was a tendency of higher pT in cohort 1 and lower pT in cohort 4. Distal subtotal gastrectomy and total gastrectomy were the major operation performed on study population (1,673 (72.5%) and 499 (21.6%), respectively), followed by proximal gastrectomy, pylorus preserving gastrectomy, near total gastrectomy and remnant total gastrectomy. The median numbers (range) of total examined LNs were 28 (6–97) in cohort 1, 37 (8–120) in cohort 2, 48 (7–122) in cohort 3, and 54 (4–221) in cohort 4. The median numbers of negative LNs was 24 (0–74) in cohort 1, 34 (2–120) in cohort 2, 44 (0–118) in cohort 3, and 51 (1–221) in cohort 4. The four cohorts significantly differed from each other regarding the total number of LNs (p < 0.001; Fig 1A) and the number of negative LNs (p < 0.001; Fig 1B); more recent cohorts (cohort 3 and 4) showed greater numbers of total and negative LNs. In addition, we have found that the total number of examined LNs were significantly different according to the operation types by Kruskall-Wallis test in all cohorts (p < 0.001). (S1 Table).

**Table 1:** Clinicopathologic characteristics of four cohorts.

	Total	Cohort 1	Cohort 2	Cohort 3	Cohort 4	*p*-value
Gender						0.004
Male	1575 (68.2%)	485 (73.0%)	384 (66.3%)	402 (68.5%)	304 (63.47%)	
Female	734 (31.8%)	179 (27.0%)	195 (33.7%)	185 (31.5%)	175 (36.53%)	
Age						<0.001^a^
Median (range)	61 (23–89)	59 (24–86)	62 (25–89)	61 (23–89)	62 (27–88)	
Mean ± SD	59.79 ± 12.41	57.61 ± 12.11	59.89 ± 11.81	60.68 ± 12.93	61.57 ± 12.47	
pT stage						<0.001 ^a^
pT1	1227 (53.2%)	311 (46.8%)	299 (51.6%)	318 (54.2%)	299 (62.4%)	
pT2	254 (11.0%)	80 (12.1%)	62 (10.7%)	70 (11.9%)	42 (8.8%)	
pT3	437 (18.9%)	168 (25.3%)	104 (18.0%)	97 (16.5%)	68 (14.2%)	
pT4	391 (16.9%)	105 (15.8%)	114 (19.7%)	102 (17.4%)	70 (14.6%)	
Operation						<0.001 ^a^
DSG	1673 (72.5%)	451 (67.9%)	453 (78.2%)	433 (73.8%)	336 (70.1%)	
TG	499 (21.6%)	190 (28.6%)	109 (18.8%)	107 (18.2%)	93 (19.4%)	
Near TG	15 (0.6%)	2 (0.3%)	3 (0.5%)	7 (1.2%)	3 (0.6%)	
PG	77 (3.3%)	1 (2.6%)	4 (0.7%)	25 (4.3%)	31 (6.5%)	
PPG	25 (1.1%)	3 (0.5%)	10 (1.7%)	10 (1.7%)	2 (0.4%)	
Remnant TG	9 (0.4%)	0 (0.0%)	0 (0.0%)	4 (0.7%)	5 (1.0%)	
Others	11 (0.5%)	1 (0.2%)	0 (0.0%)	1 (0.2%)	9 (1.9%)	
Total no. of						<0.001 ^a^
retrieved LNs						
Median (range)	40 (4–221)	28 (6–97)	37 (8–120)	48 (7–122)	54 (4–221)	
Mean ± SD	43.52 ± 20.73	30.11 ± 12.98	41.14 ± 16.99	50.40 ± 18.98	55.56 ± 23.78	
No. of						<0.001 ^a^
negative LNs						
Median (range)	36 (0–221)	24 (0–74)	34 (2–120)	44 (0–118)	51 (1–221)	
Mean ± SD	39.51 ± 20.54	25.97 ± 12.78	36.80 ± 16.04	46.03 ± 18.47	53.52 ± 23.82	
No. of						0.001
metastatic LNs						
Median (range)	0 (0–102)	0 (0–59)	0 (0–95)	0 (0–71)	0 (0–102)	
Mean ± SD	4.00 ± 8.93	4.13 ± 7.85	4.35 ± 9.54	4.32 ± 9.58	3.03 ± 8.72	
Ratio						<0.001 ^a^
Median (range)	0 (0–1.00)	0 (0–1.00)	0 (0–0.95)	0 (0–1.00)	0 (0–0.99)	
Mean ± SD	0.09 ± 0.18	0.13 ± 0.22	0.09 ± 0.18	0.08 ± 0.16	0.05 ± 0.12	
LODDS						<0.001 ^a^
Median (range)	-1.67	-1.51	-1.65	-1.77	-1.88	
	(-2.65–2.14)	(-2.17–1.76)	(-2.38–1.22)	(-2.34–2.14)	(-2.65–1.83)	
Mean± SD	-1.39 ± 0.72	-1.18 ± 0.76	-1.35 ± 0.70	-1.48 ± 0.70	-1.64 ± 0.62	
Total	2,309	664	579	587	479	

*SD* standard deviation, *DSG* distal subtotal gastrectomy, *TG* total gastrectomy, *PG* proximal gastrectomy, *PPG* pylorus preserving gastrectomy, LN lymph node

^a^ *p*-value < 0.5 is considered statistically significant

Regarding the number of metastatic LNs, the cohort 1 to 3 did not significantly differ from each other (p > 0.05 between cohort 1 and 2, 1 and 3, and 2 and 3 by Mann Whitney U tests; data not shown), but the cohort 4 had the lowest number of metastatic LNs (p < 0.001 between cohort 1 and 4, 2 and 4, and 3 and 4 by Mann Whitney U tests; Fig 1C). Additionally, cohort 4 had the lower pT (p < 0.001). The MLR and LODDS were the highest in cohort 1 and the lowest in cohort 4 (Fig 1D and 1E; p < 0.001).

### N categories from four cohorts within each pT

The four cohorts showed different clinicopathological features, especially pT category. We compared the numbers of total, negative, metastatic LNs, MLR and LODDS values from the four cohorts within each pT category. The numbers of total LNs in four cohorts were significantly different (p < 0.001 in pT1 to pT4), and the numbers of negative LNs increased along with the increase of total LNs (p < 0.001 in pT1 to pT4). The number of metastatic LNs and the MLR values from each cohort did not show a significant difference in pT1 and pT2 categories, most-likely due to the very small mean number of metastatic LNs (only 0.26 in pT1 stage and 1.20 in pT2 stage) (S2 Table). However, the MLR and LODDS values from each cohort were significantly different in the pT3 and pT4 categories (p < 0.001), and the MLR and LODDS values in each cohort showed decreasing tendency as the numbers of total LNs increased (Table 2). The number of metastatic LNs from each cohort did not show a significant difference in pT3 and pT4 categories, although significant difference was noted in the total number of LNs.

**Table 2:** Comparison of three pN categories within pT3 and pT4 cases.

		Total ^a^	Cohort 1 ^a^	Cohort 2 ^a^	Cohort 3 ^a^	Cohort 4 ^a^	*p*-value
pT3	Total LNs	46.32 ± 22.43	33.42 ± 14.09	45.02 ± 17.72	58.41 ± 19.46	62.91 ± 29.04	<0.001^b^
	Negative LNs	39.89 ± 22.71	26.26 ± 13.34	38.36 ± 17.56	52.38 ± 19.11	58.12 ± 29.72	<0.001^b^
	Metastatic LNs	6.42 ± 8.32	7.15 ± 8.78	6.66 ± 8.20	6.03 ± 8.35	4.81 ± 7.10	0.188
	Ratio	0.15 ± 0.19	0.21 ± 0.23	0.15 ± 0.19	0.10 ± 0.14	0.09 ± 0.12	<0.001^b^
	LODDS	-1.00 ± 0.70	-0.79 ± 0.72	-0.98 ± 0.66	-1.18 ± 0.61	-1.30 ± 0.63	<0.001^b^
pT4	Total LNs	49.16 ± 21.98	32.87 ± 13.40	48.63 ± 19.46	56.95 ± 20.19	63.13 ± 23.46	<0.001^b^
	Negative LNs	34.73 ± 21.65	19.87 ± 13.31	34.65 ± 18.76	39.87 ± 20.21	49.66 ± 24.62	<0.001^b^
	Metastatic LNs	14.43 ± 14.82	13.00 ± 10.70	13.98 ± 15.78	17.08 ± 15.14	13.47 ± 17.64	0.050
	Ratio	0.31 ± 0.26	0.41 ± 0.28	0.28 ± 0.25	0.30 ± 0.25	0.21 ± 0.22	<0.001^b^
	LODDS	-0.49 ± 0.74	-0.23 ± 0.76	-0.58 ± 0.72	-0.47 ± 0.67	-0.78 ± 0.70	<0.001^b^

*LN* lymph node, *LODDS* log odds of positive lymph nodes staging

^a^ All variables, mean ± standard deviation

^b^ *p*-value < 0.5 is considered statistically significant

### N categories in the cases with examined LNs of less than 16

As shown in S3 Table, 86 cases (3.87%) had total LNs of less than 16. Assessment of pT category by 7^th^ AJCC, pN category by 6^th^, 7^th^ AJCC, and MLR using three sets of cut-offs revealed that the proportion of early stage disease was higher in patients with total LNs less than 16. The MLR using the cut-off values of 0.2 and 0.5 showed the same N- category distribution as the AJCC 7^th^ edition system (R0 in 82.6%, R1 in 7.0%, R2 in 8.1%, and R3 in 2.3%). However, the N- categories by MLR using the cut-off values of 0.3 and 0.6 shifted to lower N-stages (R0 in 82.6%, R1 in 12.8%, R2 in 3.5% and R3 in 1.2%). Furthermore, most of cases with less than 16 examined LNs were categorized as LODDS1 (91.9%).

### Prediction of patients’ outcome by using each N-category model

**Fig 2:**
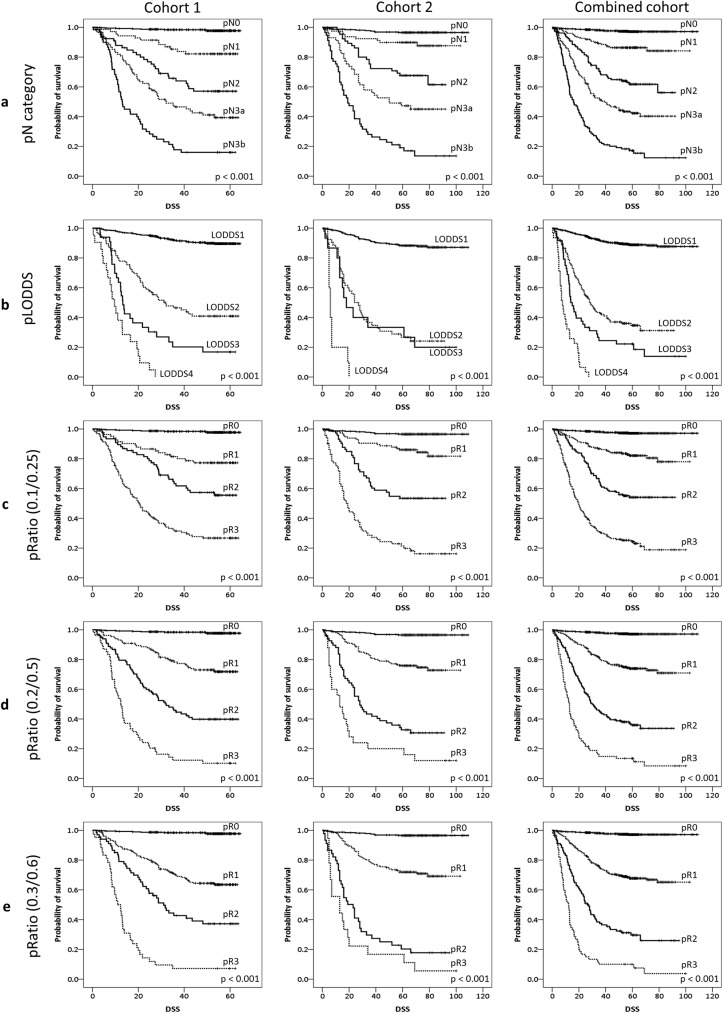
Kaplan-Meier survival curves of disease specific survival (DSS) are shown. AJCC 7^th^ N-category (a), log-odds of positive lymph node stage (LODDS) (b), and metastatic lymph node ratio (MLR) using cut-off value sets of 0.1/0.25 (c), 0.2/0.5 (d), and (0.3/0.6) (e) were all able to discriminate significant DSS differences (p < 0.001).

The Kaplan-Meier survival curves of cohort 1, cohort 2, and combined cohort 1 and 2 according to each N-category model are shown in Fig 2. Overall, all AJCC 7^th^ pN category, pLODDS, and pMLR by three sets of cut-off values were able to discriminate the DSS in cohort 1, cohort 2 and combined cohort with statistical significance (p < 0.001). One notable finding was that in cohort 2, the distinction of DSS between LODDS2 and LODDS3 was not clear (p = 0.745).

Since the Kaplan-Meier survival analysis alone could not prove which of the N-category model is the best for prediction of patients’ outcome, we adapted the AIC and BIC indices for each model, which showed distinct results depending on the cohort, as shown in Table 3. For the cohort 1, the MLR model using the cut-off values of 0.2 and 0.5 was found to be the best model. The cohort 2, which consisted of patients with a greater number of examined LNs, was best explained by the MLR model with the lower cut-off values of 0.1 and 0.25. The results were different in combined cohort: AIC favored the AJCC 7^th^ edition system, and BIC supported MLR with cut-offs of 0.2 and 0.5.

**Table 3:** AIC index and BIC index according to various pN categories.

	Cohort 1 (N = 664)					Cohort 2 (N = 579)				Combined cohort (N = 1,243)			
	N	DSS(%)	AIC	BIC	N		DSS(%)	AIC	BIC	N	DSS(%)	AIC	BIC
pN (AJCC7^th^)			1577.7	1595.1				1245.7	1263.2			3178.7†	3199.2
pN0	379	97.9			320		96.9			699	97.3		
pN1	70	82.9			79		89.9			149	86.6		
pN2	66	59.1			66		68.2			132	63.6		
pN3a	89	42.7			57		47.4			146	44.5		
pN3b	60	16.7			57		17.5			117	17.1		
pLODDS			1643.0	1656.5				1303.8	1316.9			3301.4	3316.8
pL0	523	90.2			501		88.4			1024	89.4		
pL1	87	43.7			53		28.3			140	37.9		
pL2	33	18.2			15		26.7			48	20.8		
pL3	21	0.0			10		0.0			31	0.0		
pRatio (0.1/0.25)			1590.0	1603.5				1238.4†	1251.5†			3183.7	3199.1
pR0	379	97.9			320		96.6			699	97.3		
pR1	82	78.0			115		86.1			197	82.7		
pR2	76	57.9			73		53.4			149	55.7		
pR3	127	29.1			71		21.1			198	26.3		
pRatio (0.2/0.5)			1565.4†	1578.9†				1262.3	1275.4			3180.0	3195.3†
pR0	379	97.9			320		96.6			699	97.3		
pR1	132	73.5			166		75.9			298	74.8		
pR2	99	42.4			68		33.8			167	38.9		
pR3	54	11.1			25		16.0			79	12.7		
pRatio (0.3/0.6)			1577.7	1591.2				1260.7	1273.8			3197.4	3212.8
pR0	379	97.9			320		96.6			699	97.3		
pR1	176	65.3			196		71.9			372	68.8		
pR2	67	40.3			45		22.2			112	33.0		
pR3	42	7.1			18		11.1			60	8.3		

*AIC* Akaike information criterion, *BIC* Bayesian information criterion, *DSS* disease specific survival, *AJCC* American Joint Committee on Cancer, *LODDS* log odds of positive lymph nodes

† On interpretation of AIC and BIC indices, the models with lowest value are preferred

## Discussion

In this study, we compared the numbers of total, negative, and metastatic LNs in four independent cohorts to clarify clinical significance of maximal pathologic evaluation of LNs. The numbers of examined LNs of our study far exceeded those of previous studies from various institutions [11,12,19]. Based on the results of previous studies on this topic, we have expected that increased number of examined LNs would probably result in an increased number of positive LNs [12,13]. However, the number of negative LNs increased with the increase of total LN, but the number of metastatic LNs did not.

In addition, using the DSS of two cohorts, we aimed to identify the pN category model with best prognostic performance. With the exception of the AIC index for the combined total population, all the indices supported the MLR system as the best pN category model. However, there are some points to consider when applying MLR. It is mathematically dependent on the total number of examined LNs; our results showed that the proportion of patients with advanced N-category decreases as the total number of examined LNs increases from the cohort 1 to 4, and it suggests the possibility of understaging, due to an intrinsic flaw in the MLR calculation. Also, our data showed that this phenomenon of understaging by MLR is more prominent in patients with advanced pT stage, in contrast to the absolute number of metastatic LNs. In addition, we have found that total number of LNs vary according to the operation types, which may result in confounder of MLR or LODD system.

Also, there is no single consensus regarding the cut-off values in MLR. Different cut-off values—more than ten sets to our knowledge—were applied in previous studies on MLR (Table 4) [13,17,23–31]. When higher cut-off values were applied (e.g. the set of 0.3 and 0.6 rather than the set of 0.1 and 0.25), the tendency of understaging would be intensified. In the cohort 2 of our study, in which more LNs were examined than in the cohort 1, the model using the lower cut-off values— 0.1 and 0.25 —turned out the be the best according to the AIC and BIC indices, while the model using 0.2 and 0.5 was the best for the cohort 1. From these findings, we suggest that the superiority of the MLR system over the AJCC 7^th^ edition system is questionable, for it may be influenced by population characteristics and cut-off values.

**Table 4:** Variable cut-off values for metastatic lymph node ratio (MLR) system in previous studies.

Author	Year	Population	N	AJCC	MLR cut-off	Result
Koh	Current study	Korea; multicenter	2,309	7^th^	0.1 and 0.25	MLR not superior
					0.2 and 0.5	
					0.3 and 0.6	
Lorenzon L	2014	Italy;single center	129	6^th^, 7^th^	0.01, 0.05, 0.10, 0.20, and 0.30	Favor MLR
Alatengbaolide	2013	China;single center	1,916	5^th^	0.1 and 0.25	Favor MLR
Aurello P	2013	Italy;single center	142	7^th^	0.1 and 0.25	Favor MLR
Wong J	2013	US;single center	222	7^th^	0.2 and 0.5	Favor MLR
Nelen SD	2013	Netherland; multicenter	973	5^th^, 6^th^, 7^th^	0.1, 0.20, and 0.3	Favor MLR
Zeng WJ	2013	China; multicenter	613	7^th^	0.5 and 0.8	Favor MLR
Xu J	2013	China;single center	427	7^th^	0.2, 0.4, and 0.7	MLR not superior
Kong SH	2012	Korea;single center	8,949	7^th^	0.2 and 0.5	Favor MLR
Lee SR	2012	Korea;single center	495	6^th^	0.01, 0.05, 0.10, 0.20, and 0.30	Favor MLR
Wang J	2012	US;multicenter	18,043	7^th^	1/15, 3/10, and 7/10	Favor MLR
Xiao LB	2011	China;single center	1,042	6^th^, 7^th^	0.01, 0.30, and 0.50	Favor MLR
Bilici A	2010	Turkey;single center	202	5^th^	0.1 and 0.25	MLR not superior

*N* number of cases, *AJCC* American Joint Committee on Cancer, *MLR* metastatic lymph node ratio

The major advantage of LODDS compared to number-based AJCC 7^th^ pN-category or MLR was that only LODDS can discriminate survival differences within the pN0 category. However, in our study population all the pN0 patients in all four cohorts were in LODDS1 (S4 Table), most likely owing to mathematical calculation using the large number of total harvested lymph nodes in our cohorts. Therefore, we inferred that LODDS have limitation in further discrimination of pN0 category, especially when the total number of lymph nodes are high.

The AJCC 7^th^ edition system has its own superiorities. This number-based LN staging system has long been the most popular way of assessing node status. It is widely used in various types of cancer, and it is the simplest and most familiar way for both pathologists and clinicians. It is also straightforward, while MLR and LODDS take additional mathematical calculation. Our results shown in Table 3 suggest that the prognostic performance of AJCC 7^th^ edition method was not inferior to the MLR system when the cohorts were combined.

Owing to the fact that the total number of retrieved LN varies from region to region [32], one of possible disadvantages when using the MLR is understaging of N-category, particularly in cases with extended LN dissection. Although there have been debates regarding the risk and benefit of D2 dissection in gastric cancer, there is a considerable evidence for the survival benefit of extended lymphadenectomy in the Asian population [33–35]. Additionally, some authors have suggested that insufficient lymphadenectomy may be associated with increased locoregional recurrence [7]. If the surgical procedure is standardized, the number of total examined LNs would depend on the procedure of handling and identifying LNs in pathologic laboratories. The pathologist or pathologists’ assistant inspects and palpates the perigastric fat to identify LNs in daily practice, which is a very labor-intensive and time-consuming procedure. Our results showed that the number of metastatic LNs did not increase with the increase of total LN number, thus more thorough examination of LNs in pathologic laboratories might not be meaningful if total retrieved LNs are above a certain amount.

This study has a limitation of being a retrospective study based on a Korean population. In addition, since we enrolled all consecutively registered GC patients in two institutes from year 2003 to 2013, adjuvant chemotherapy could not be controlled. However, the patients with stage II to IV disease received adjuvant chemotherapy using fluoropyrimidine-based regimens. This un-controlled adjuvant chemotherapy may have acted as the confounder of the survival results of the study population. Therefore, generalization of our results to various patients with gastric cancer worldwide may not be feasible. Additionally, since most of the patients in this study population had undergone D2 dissection, further international multi-center studies or validation in other regions, where the conservative lymphadenectomy is relatively common, is required.

In summary, our results show that the increase of the number of total examined LNs does not always result in the increased metastatic LNs. Therefore, we suggest that more meticulous LN sampling in pathologic laboratories is not always necessary for optimal pN category, if total LNs are above a certain amount. In addition, MLR and LODDS values were influenced by the number of total LNs. However, the AJCC 7^th^ edition system gave relatively consistent results with a single consensus on the cut-off value. For the MLR system to become more reliable, we should be aware of its limitations, especially the issues regarding the cut-off values.

## Supporting information

S1 Table The number of total examined lymph nodes according to various operation types. (DOCX)

S2 Table Comparison of three N category methods within pT1 and pT2 cases. (DOCX)

S3 Table Characteristics of population with less than 16 examined lymph nodes. (DOCX)

S4 Table Comparison of AJCC 7^th^ system and LODDS. (DOCX)

S5 Table The results of Cox proporational hazard model in cohort 1 and cohort 2. (DOCX)

S1 File Dataset including the key clinicopathologic features of study population. (XLSX)
