# Supplementary material for: Clinicopathologic implication of meticulous pathologic examination of regional lymph nodes in gastric cancer patients
Source: PLoS One. 2017 Mar 31;12(3):e0174814. doi: 10.1371/journal.pone.0174814 (PMC5376083; doi:10.1371/journal.pone.0174814)
Supplement: S1 Table — (DOCX) [file pone.0174814.s001.docx]

S1 Table. The number of total examined lymph nodes according to various operation types

|  | Total | Cohort 1 | Cohort 2 | Cohort 3 | Cohort 4 |
| --- | --- | --- | --- | --- | --- |
| DSG | 39  (5 – 156) | 27  (7 – 72) | 36  (8 – 120) | 47  (7 – 110) | 53  (5 – 156) |
| TG | 44  (4 – 221) | 32  (6 – 97) | 44  (19 – 102) | 58  (13 – 122) | 66  (4 – 221) |
| Near TG | 47  (16 – 143) | 48  (39 – 56) | 45  (16 – 48) | 47  (37 – 111) | 67  (21 – 143) |
| PG | 35  (11 – 89) | 22  (11 – 75) | 29  (19 – 74) | 37  (20 – 61) | 41  (18 – 68) |
| PPG | 31  (13 – 64) | 31  (27 – 40) | 31  (13 – 46) | 36  (13 – 64) | 34  (26 – 42) |
| Remnant TG | 22  (4 – 36) | - | - | 19  (14 – 24) | 22  (4 – 36) |
| Others | 42  (10 – 85) | 55  (55 – 55) | - | 50  (50 – 50) | 34  (10 – 85) |

*DSG* distal subtotal gastrectomy, *TG* total gastrectomy, *PG* proximal gastrectomy, *PPG* pylorus preserving gastrectomy

* All numeric data are shown as median (range)
