# Supplementary material for: Clinicopathologic implication of meticulous pathologic examination of regional lymph nodes in gastric cancer patients
Source: PLoS One. 2017 Mar 31;12(3):e0174814. doi: 10.1371/journal.pone.0174814 (PMC5376083; doi:10.1371/journal.pone.0174814)
Supplement: S2 Table — (DOCX) [file pone.0174814.s002.docx]

S2 Table. Comparison of three N category methods within pT1 and pT2 cases

|  |  | Cohort 1^a^ | Cohort 2 ^a^ | Cohort 3 ^a^ | Cohort 4 ^a^ | Total | *p*-value |
| --- | --- | --- | --- | --- | --- | --- | --- |
| pT1 | Total no. of  retrieved LNs | 27.01  ± 11.32 | 36.53  ± 13.54 | 46.20  ± 17.00 | 53.55  ± 21.90 | 40.77  ± 19.19 | <0.001^b^ |
|  | No. of  negative LNs | 26.75  ± 11.35 | 36.15  ± 13.38 | 45.94  ± 17.03 | 53.19  ± 21.91 | 40.46  ± 19.17 | <0.001 ^b^ |
|  | No. of  metastatic LNs | 0.26  ± 1.38 | 0.38  ± 1.42 | 0.26  ± 1.04 | 0.36  ± 1.98 | 0.31  ± 1.49 | 0.087 |
|  | Ratio  (mean ± SD) | 0.0098  ± 0.0488 | 0.0095  ± 0.0314 | 0.0060  ± 0.0246 | 0.0070  ± 0.0399 | 0.0080  ± 0.0373 | 0.084 |
|  | LODDS  (mean ± SD) | -1.6371  ± 0.2950 | -1.7384  ± 0.2993 | -1.8629  ± 0.2783 | -1.9180  ± 0.3213 | -1.7888  ± 0.3176 | <0.001 ^b^ |
| pT2 | Total no. of  retrieved LNs | 31.56  ± 13.49 | 43.13  ± 19.23 | 48.84  ± 19.22 | 56.64  ± 24.04 | 43.30  ± 20.61 | <0.001 ^b^ |
|  | No. of  negative LNs | 30.36  ± 13.56 | 41.26  ± 18.85 | 47.06  ± 18.61 | 54.88  ± 24.45 | 41.68  ± 20.29 | <0.001 ^b^ |
|  | No. of  metastatic LNs | 1.20  ± 2.19 | 1.87  ± 3.51 | 1.79  ± 3.75 | 1.76  ± 2.45 | 1.62  ± 3.06 | 0.411 |
|  | Ratio  (mean ± SD) | 0.0413  ± 0.0734 | 0.0424  ± 0.0700 | 0.0393  ± 0.0737 | 0.0338  ± 0.0466 | 0.0398  ± 0.0686 | 0.770 |
|  | LODDS  (mean ± SD) | -1.4518  ± 0.4720 | -1.4797  ± 0.4805 | -1.5898  ± 0.5099 | -1.5915  ± 0.5009 | -1.5197  ± 0.4908 | 0.088 |

*LN* lymph node, *SD* standard deviation

^a^ All variables, mean ± standard deviation

^b^ *p*-value < 0.5 is considered statistically significant
