# Supplementary material for: Clinicopathologic implication of meticulous pathologic examination of regional lymph nodes in gastric cancer patients
Source: PLoS One. 2017 Mar 31;12(3):e0174814. doi: 10.1371/journal.pone.0174814 (PMC5376083; doi:10.1371/journal.pone.0174814)
Supplement: S3 Table — (DOCX) [file pone.0174814.s003.docx]

S3 Table. Characteristics of population with less than 16 examined lymph nodes

|  |  | No. of total LNs | | p value |
| --- | --- | --- | --- | --- |
|  |  | less than 16 | 16 or more |  |
| pT |  |  |  | 0.001^a^ |
|  | pT1 | 63 (73.3%) | 1164 (52.4%) |  |
|  | pT2 | 8 (9.3%) | 246 (11.1%) |  |
|  | pT3 | 8 (9.3%) | 429 (19.3%) |  |
|  | pT4 | 7 (8.1%) | 384 (17.3%) |  |
| LODDS |  |  |  | 0.492 |
|  | LODDS1 | 79 (91.9%) | 1916 (86.2%) |  |
|  | LODDS2 | 5 (5.8%) | 198 (8.9%) |  |
|  | LODDS3 | 1 (1.2%) | 67 (3.0%) |  |
|  | LODDS4 | 1 (1.2%) | 42 (1.9%) |  |
| pRatio (0.1/0.25) |  |  |  |  |
|  | R0 | 71 (82.6%) | 1284 (57.8%) | <0.001^a^ |
|  | R1 | 5 (5.8%) | 409 (18.4%) |  |
|  | R2 | 5 (5.8%) | 254 (11.4%) |  |
|  | R3 | 5 (5.8%) | 276 (12.4%) |  |
| pRatio (0.2/0.5) |  |  |  |  |
|  | R0 | 71 (82.6%) | 1284 (57.8%) | <0.001^a^ |
|  | R1 | 6 (7.0%) | 588 (26.5%) |  |
|  | R2 | 7 (8.1%) | 242 (10.9%) |  |
|  | R3 | 2 (2.3%) | 109 (4.9%) |  |
| pRatio (0.3/0.6) |  |  |  |  |
|  | R0 | 71 (82.6%) | 1284 (57.8%) | <0.001^a^ |
|  | R1 | 11 (12.8%) | 700 (31.5%) |  |
|  | R2 | 3 (3.5%) | 162 (7.3%) |  |
|  | R3 | 1 (1.2%) | 77 (3.5%) |  |
| pN (7^th^) |  |  |  |  |
|  | pN0 | 71 (82.6%) | 1284 (57.8%) | <0.001^a^ |
|  | pN1 | 6 (7.0%) | 276 (12.4%) |  |
|  | pN2 | 7 (8.1%) | 231 (10.4%) |  |
|  | pN3a | 2 (2.3%) | 239 (10.8%) |  |
|  | pN3b | 0 (0%) | 193 (8.7%) |  |
| Total |  | 86 (3.7%) | 2223 (96.3%) |  |

*LN* lymph node, *LODDS* log odds of positive lymph nodes staging

^a^*p*-value < 0.5 is considered statistically significant
