# Supplementary material for: Clinicopathologic implication of meticulous pathologic examination of regional lymph nodes in gastric cancer patients
Source: PLoS One. 2017 Mar 31;12(3):e0174814. doi: 10.1371/journal.pone.0174814 (PMC5376083; doi:10.1371/journal.pone.0174814)
Supplement: S4 Table — (DOCX) [file pone.0174814.s004.docx]

S4 Table. Comparion of AJCC 7^th^ system and LODDS

|  | pN0 | pN1 | pN2 | pN3a | pN3b | Total |
| --- | --- | --- | --- | --- | --- | --- |
| LODDS  LODDS1  LODDS2  LODDS3  LODDS4 | 1355 (100.0%)  0 (0.0%)  0 (0.0%)  0 (0.0%) | 282 (100.0%)  0 (0.0%)  0 (0.0%)  0 (0.0%) | 220 (92.4%)  18 (7.6%)  0 (0.0%)  0 (0.0%) | 127 (52.7%)  99 (41.1%)  12 (5.0%)  3 (1.2%) | 11 (5.7%)  86 (44.6%)  56 (29.0%)  40 (20.7%) | 1995 (86.4%)  203 (8.8%)  68 (2.9%)  43 (1.9%) |
| Total | 1355 (58.7%) | 282 (12.2%) | 238 (10.3%) | 241 (10.4%) | 193 (8.4%) | 2309 (100.0%) |
