# Supplementary material for: Clinicopathologic implication of meticulous pathologic examination of regional lymph nodes in gastric cancer patients
Source: PLoS One. 2017 Mar 31;12(3):e0174814. doi: 10.1371/journal.pone.0174814 (PMC5376083; doi:10.1371/journal.pone.0174814)
Supplement: S5 Table — (DOCX) [file pone.0174814.s005.docx]

S5 Table. The results of Cox proporational hazard model in cohort 1 and cohort 2.

|  | Total population | | Cohort 1 | | Cohort 2 | |
| --- | --- | --- | --- | --- | --- | --- |
|  | Beta | HR (95% CI) | Beta | HR (95% CI) | Beta | HR (95% CI) |
| pN  (AJCC7th) |  |  |  |  |  |  |
| pN0 | - | - | - | - | - | - |
| pN1 | 1.648 | 5.20 (2.77-9.74) | 2.161 | 8.68 (3.55-21.24) | 1.114 | 3.05 (1.23-7.58) |
| pN2 | 2.781 | 16.14 (9.49-27.47) | 3.191 | 24.32 (11.05-53.55) | 2.371 | 10.71 (5.16-22.21) |
| pN3a | 3.395 | 29.80 (18.07-49.17) | 3.706 | 40.71 (19.30-85.90) | 3.046 | 21.02 (10.52-42.00) |
| pN3b | 4.183 | 65.56 (39.95-107.61) | 4.489 | 88.99 (41.96-188.75) | 3.899 | 49.34 (25.45-95.64) |
| pLODDS |  |  |  |  |  |  |
| pL0 | - | - | - | - | - | - |
| pL1 | 2.228 | 9.28 (6.98-12.34) | 2.153 | 8.61 (5.80-12.77) | 2.366 | 10.65 (7.04-16.11) |
| pL2 | 2.734 | 15.39 (10.59-22.36) | 2.888 | 17.96 (11.17-28.86) | 2.451 | 11.60 (6.07-22.16) |
| pL3 | 3.848 | 46.91 (30.61-71.89) | 3.749 | 42.48 (24.71-73.01) | 4.111 | 60.99 (29.39-126.58) |
| pRatio  (0.1/0.25) |  |  |  |  |  |  |
| pR0 | - | - | - | - | - | - |
| pR1 | 1.914 | 6.78 (3.87-11.88) | 2.447 | 11.56 (5.03-26.59) | 1.439 | 4.22 (1.96-9.09) |
| pR2 | 3.011 | 20.31 (12.18-33.85) | 3.199 | 24.50 (11.29-53.20) | 2.834 | 17.01 (8.61-33.60) |
| pR3 | 3.972 | 53.10 (32.82-85.89) | 4.117 | 61.38 (29.70-126.86) | 3.874 | 48.15 (25.10-92.37) |
| pRatio  (0.2/0.5) |  |  |  |  |  |  |
| pR0 | - | - | - | - | - | - |
| pR1 | 2.328 | 10.25 (6.20-16.97) | 2.649 | 14.13 (6.56-30.47) | 2.038 | 7.68 (3.94-14.96) |
| pR2 | 3.563 | 35.25 (21.57-57.62) | 3.721 | 41.30 (19.68-86.69) | 3.435 | 31.03 (16.01-60.14) |
| pR3 | 4.476 | 87.89 (52.59-146.90) | 4.736 | 113.98 (53.47-242.95) | 4.158 | 63.92 (30.63-133.42) |
| pRatio  (0.3/0/6) |  |  |  |  |  |  |
| pR0 | - | - | - | - | - | - |
| pR1 | 2.589 | 13.32 (8.20-21.64) | 2.986 | 19.81 (9.48-41.41) | 2.216 | 9.17 (4.80-17.53) |
| pR2 | 3.746 | 42.35 (25.55-70.20) | 3.767 | 43.25 (20.21-92.56) | 3.829 | 46.03 (23.27-91.06) |
| pR3 | 4.668 | 106.51 (62.71-180.90) | 4.951 | 141.35 (65.25-306.19) | 4.296 | 73.39 (33.80-159.35) |

*Beta* Beta-coefficient, *HR* hazard ratio, *CI* confidence interval
